# Association between the C-reactive protein-triglyceride-glucose index and major adverse cardiovascular events in patients undergoing percutaneous coronary intervention

**DOI:** 10.3389/fmed.2026.1858059

**Published:** 2026-05-29

**Authors:** Feilong Shao, Yazhao Sun, Chunlan Bai

**Affiliations:** 1 Department of General Practice, Huabei Petroleum Administration Bureau General Hospital, Cangzhou, Hebei, China; 2 Department of Cardiology, Cangzhou People's Hospital, Cangzhou, Hebei, China

**Keywords:** C-reactive protein–triglyceride-glucose index, inflammation, insulin resistance, major adverse cardiovascular events, percutaneous coronary intervention

## Abstract

**Background:**

The interaction between inflammation and insulin resistance is a key driver of atherosclerosis. However, the combined effect of C-reactive protein (CRP) and triglyceride-glucose index (TyG) on the risk of major adverse cardiovascular events (MACEs) remains unclear. This study aims to evaluate the association between the C-reactive protein-triglyceride-glucose index (CTI) and major adverse cardiovascular events following percutaneous coronary intervention (PCI).

**Methods:**

Retrospective data from 2,610 patients who underwent PCI were collected. The association between the CTI and the risk of adverse outcomes was evaluated using survival analysis, Cox regression, restricted cubic splines (RCS), time-dependent receiver operating characteristic (ROC) curves, subgroup analysis, and sensitivity analysis.

**Results:**

During the follow-up period, 459 participants experienced MACEs. The incidence of adverse outcomes differed significantly across CTI levels, and Cox regression analysis indicated that CTI is an independent predictor. RCS analysis showed a non-linear relationship between CTI and the primary endpoint, and a linear relationship with the secondary endpoint. CTI demonstrated good predictive ability for adverse outcomes, and the results were validated by subgroup and sensitivity analyses, confirming their stability.

**Conclusion:**

Pre-PCI CTI levels are significantly associated with adverse outcome.

## Introduction

Coronary artery disease (CAD) is a leading cause of death and long-term disability worldwide, placing considerable strain on public health systems. Despite significant advances in the treatment of CAD through percutaneous coronary intervention (PCI), there remains a high incidence of major adverse cardiovascular events following the procedure (1). However, due to differences in demographic characteristics, medical history, blood biomarkers, coronary angiographic findings, and medication regimens, patients with CAD undergoing percutaneous coronary intervention exhibit significant heterogeneity in postoperative outcomes. Previous studies have identified several factors influencing the occurrence of major adverse cardiovascular events after percutaneous coronary intervention, including age, type 2 diabetes, hypertension, chronic kidney disease, elevated low-density lipoprotein cholesterol (LDL-C) levels, eccentric lesions (atherosclerotic plaques that occupy only a portion of the vessel circumference, resulting in uneven stress distribution and a higher risk of plaque rupture), multivessel disease, and the number of stents implanted (2). The ultimate goal of percutaneous coronary intervention is not only to prolong survival but also to improve prognosis and quality of life (3). Therefore, identifying patients who undergo percutaneous coronary intervention but remain at high cardiovascular risk is crucial for optimizing postoperative management and reducing the incidence of adverse cardiovascular events.

Insulin resistance is a pathological condition characterized by a decreased sensitivity of the body to insulin, serving as a core mechanism in various metabolic diseases and a key factor in atherosclerotic cardiovascular disease. In clinical practice, the triglyceride-glucose index (TyG index), which is calculated from fasting blood glucose (FBG) and fasting triglycerides (TG), has emerged as a reliable and novel indicator for assessing insulin resistance (4). The TyG index correlates with the normal blood glucose-high insulin clamp test, and its effectiveness is comparable to the homeostasis model assessment of insulin resistance (5). Kurniawan et al. found that a higher TyG index was significantly associated with the incidence of type 2 diabetes (6). Additionally, insulin resistance increases the risk of atherosclerotic cardiovascular disease through mechanisms such as lipotoxicity, glucotoxicity, chronic low-grade inflammation, oxidative stress, and endothelial dysfunction (7, 8). Several studies have highlighted the significant association between the TyG index and hypertension, CAD, and intermediate cardiovascular risk (9–11). However, Yang et al. noted that the TyG index is not an effective predictor of adverse cardiovascular outcomes in non-diabetic patients undergoing PCI (12). Meanwhile, inflammation plays a critical role in the pathogenesis of cardiovascular diseases. Chronic low-grade inflammation is considered a key factor in atherosclerotic plaque formation, plaque instability, and endothelial dysfunction, all of which significantly increase the risk of cardiovascular events. C-reactive protein (CRP), an acute-phase protein, responds rapidly to inflammation and infection, and its role in cardiovascular diseases has been extensively studied. Research has shown that CRP is not merely a passive marker of inflammation but may also play an active role in the development and progression of atherosclerosis (13). Furthermore, CRP not only serves as a predictor of cardiovascular events but can also be used to monitor disease severity and progression. For instance, in patients with CAD, CRP levels can independently predict the severity of coronary artery stenosis, regardless of LDL-C levels (14). Therefore, developing comprehensive biomarkers that reflect both insulin resistance and inflammatory status is crucial for predicting cardiovascular disease risk. The C-reactive protein-triglyceride-glucose index (CTI), proposed by Ruan et al. in 2022 (15), is a novel, economical, and easily accessible biochemical marker that combines CRP and the TyG index to assess both inflammation and insulin resistance severity (16). Although the predictive value of CTI in several diseases has been partially validated, its relationship with major adverse cardiovascular events (MACEs) after PCI has yet to be fully elucidated. To fill this research gap, we conducted a retrospective analysis of relevant datasets, aiming to explore the association between CTI and MACEs and evaluate its potential application value in postoperative risk prediction.

## Methods

### Study population and ethics statement

This study is a retrospective cohort study that evaluated the clinical data of 2,610 patients with CAD who underwent PCI between January 2022 and August 2022. The data were consecutively collected from the hospital information system of Cangzhou People’s Hospital. The inclusion criteria were: patients aged 18 to 80 years who underwent PCI treatment. The exclusion criteria included: (1) acute myocardial infarction; (2) history of coronary artery bypass grafting; (3) persistent or recent acute/chronic infection; (4) thyroid disease, autoimmune diseases, or hematological disorders; (5) severe heart, liver, or kidney dysfunction; (6) malignant tumors; (7) missing essential laboratory data, such as baseline lipid profile, CRP, TG, and FBG; (8) incomplete clinical information or missing follow-up data. All included patients provided written informed consent, confirming their agreement to have their medical data used for this research analysis, with appropriate measures taken to protect patient privacy during data analysis. The study protocol was approved by the Ethics Committee of Cangzhou People’s Hospital and complied with the ethical standards set forth in the Declaration of Helsinki. Based on the occurrence of MACEs during a 3-year follow-up period, patients were divided into the Non-MACEs group (*n* = 2,151) and the MACEs group (*n* = 459) for analysis.

### Exposure and outcome

CTI was calculated using the following formula (15): CTI = 0.412 × Ln (CRP [mg/L]) + Ln (TG [mg/dL] × FBG [mg/dL])/2. In the preliminary analysis, CTI was treated as a continuous variable and later categorized into quartiles for subsequent analysis.

Clinical follow-up was conducted by experienced clinicians via outpatient visits or telephone calls, with a follow-up duration of 3 years. All data were recorded in a standardized electronic case report form. The primary endpoint of the study was the first occurrence of any of the following events during the follow-up period: All-cause mortality, including sudden death and other deaths of unknown cause. Non-fatal myocardial infarction, characterized by ischemic symptoms accompanied by elevated cardiac biomarkers, ST-segment changes, or pathological Q waves, but without leading to death. Unplanned revascularization, defined as a revascularization procedure (including but not limited to PCI or coronary artery bypass grafting) driven by ischemic symptoms or objective evidence of ischemia. Hospitalization due to angina, defined as an unplanned hospital admission for worsening anginal symptoms that could not be controlled by medication. This endpoint excluded cases in which the angina episode was attributable to acute myocardial infarction (based on biomarker elevation or Q-wave changes) or in which revascularization was performed during the hospitalization, as these clinical scenarios were already captured under the non-fatal myocardial infarction or unplanned revascularization endpoints, respectively.

The secondary endpoint was a composite of all-cause mortality, non-fatal myocardial infarction, and unplanned revascularization. For the timing of events in the composite endpoint, the time interval from baseline assessment to the first event was calculated. In cases where multiple events occurred simultaneously, the earliest event was considered the endpoint for statistical analysis. In the subsequent statistical analysis, the primary endpoint will be referred to as MACEs, and the secondary endpoint will be referred to as MACE. This distinction facilitates data analysis and presentation of results.

### Covariates

This study collected various patient data through a review of medical records, which included demographic information, medical history, laboratory measurements, coronary angiographic findings, and discharge medications. The demographic data encompassed age, sex, body mass index (BMI), and smoking status. Medical history included previous stroke, previous CAD, previous PCI, hypertension, diabetes, chronic kidney disease, peripheral artery disease, and heart failure. Laboratory measurements included FBG, glycated hemoglobin (HbA1c), TG, total cholesterol (TC), LDL-C, high-density lipoprotein cholesterol (HDL-C), and CRP. Coronary angiographic findings included left main disease, multivessel disease, and chronic total occlusion. Discharge medications included antiplatelet agents, *β*-blockers, angiotensin-converting enzyme inhibitors/angiotensin receptor blocker (ACEI/ARB), and statins. BMI was calculated as weight (in kilograms) divided by the square of height (in meters).

### Statistical analysis

The main characteristics of the sample were analyzed using descriptive statistics. The Shapiro–Wilk test was used to assess the normality of continuous variables, with non-normally distributed data presented as median (interquartile range, IQR) and compared using the Mann–Whitney U test. Categorical variables were expressed as frequency (n) and percentage (%) and compared using the chi-square test or Fisher’s exact test. Outcome assessments were performed using Kaplan–Meier (K–M) survival curves, with comparisons made using the log-rank test. The association between baseline CTI and outcomes was evaluated using Cox proportional hazards models, with results presented as hazard ratio (HR) and 95% confidence interval (CI). Three Cox models were adjusted as follows: Model 1: unadjusted; Model 2: adjusted for age, BMI, smoking, and diabetes; Model 3: adjusted for age, BMI, smoking, previous CAD, previous PCI, hypertension, diabetes, chronic kidney disease, peripheral artery disease, HbA1c, LDL-C, multivessel disease, chronic total occlusion, and in-stent restenosis. To assess the dose–response relationship between baseline CTI and outcomes, restricted cubic spline (RCS) analysis was conducted with four knots. The predictive performance of CTI was compared with that of CRP alone and the TyG index alone using DeLong tests for both MACEs and MACE. Additionally, the predictive value of baseline CTI for outcomes was evaluated using time-dependent receiver operating characteristic (ROC) curves, plotted at 10, 20, and 30 months. Considering the non-linear relationship between CTI and MACEs, a segmented Cox regression model was used to evaluate the impact of CTI on the risk of MACEs across different intervals. Stratified analyses were also performed to assess the relationship between baseline CTI and outcomes by subgroups, including age, sex, smoking, overweight, diabetes, and hypertension. To ensure the robustness of the results, sensitivity analyses were performed excluding the effects of smoking, previous stroke, chronic kidney disease, and peripheral artery disease. All statistical analyses were performed using R version 4.3.2, and a two-sided *p*-value of <0.05 was considered statistically significant.

## Results

### Participant characteristics

The baseline characteristics of the 2,610 participants are shown in Table 1. The median age was 66 years, and 61.3% were male. A total of 459 participants experienced MACEs. Patients in the MACEs group were significantly older and had a higher BMI. Additionally, the proportions of Smoking, Previous CAD, Previous PCI, Hypertension, Diabetes, Chronic kidney disease, and Peripheral Artery Disease were higher in the MACEs group. The levels of FBG, HbA1c, TG, TC, LDL-C, CRP, TyG index, and CTI were significantly higher in the MACEs group. Regarding CAD, the incidence of Multivessel Disease, Chronic Total Occlusion, and In-Stent Restenosis was more common in the MACEs group.

**Table 1:** Characteristics of the patients.

Variables	Total (*n* = 2,610)	Non-MACEs (*n* = 2,151)	MACEs (*n* = 459)	*p*-value
Age (years)	66 (60, 70)	65 (59, 70)	68 (65, 70)	<0.001
Male, *n* (%)	1,599 (61.3)	1,316 (61.2)	283 (61.7)	0.891
BMI (kg/m^2^)	24.81 (23.51, 26.31)	24.78 (23.44, 26.35)	24.90 (23.85, 26.15)	0.044
Smoking, *n* (%)	602 (23.1)	469 (21.8)	133 (29.0)	0.001
Previous stroke, *n* (%)	427 (16.4)	345 (16)	82 (17.9)	0.373
Previous CAD, *n* (%)	745 (28.5)	584 (27.2)	161 (35.1)	<0.001
Previous PCI, *n* (%)	379 (14.5)	285 (13.2)	94 (20.5)	<0.001
Hypertension, *n* (%)	1,647 (63.1)	1,307 (60.8)	340 (74.1)	0.001
Diabetes, *n* (%)	832 (31.9)	626 (29.1)	206 (44.9)	<0.001
Chronic kidney disease, *n* (%)	129 (4.9)	95 (4.4)	34 (7.4)	0.010
Peripheral artery disease, *n* (%)	112 (4.3)	81 (3.8)	31 (6.8)	0.006
Heart failure, *n* (%)	489 (18.7)	400 (18.6)	89 (19.4)	0.741
FBG (mmol/L)	5.49 (4.99, 6.29)	5.45 (4.97, 6.21)	5.75 (5.16, 6.98)	<0.001
HbA1c (%)	5.8 (5.6, 6.2)	5.8 (5.6, 6.1)	5.9 (5.6, 6.3)	0.007
TG (mmol/L)	1.32 (0.95, 1.91)	1.29 (0.94, 1.83)	1.55 (1.05, 2.34)	<0.001
TC (mmol/L)	4.43 (3.70, 5.19)	4.36 (3.63, 5.14)	4.71 (3.99, 5.42)	<0.001
LDL-C (mmol/L)	2.67 (2.06, 3.34)	2.63 (2.01, 3.32)	2.85 (2.24, 3.45)	<0.001
HDL-C (mmol/L)	1.21 (1.02, 1.42)	1.22 (1.02, 1.42)	1.18 (1.00, 1.42)	0.190
CRP (mg/L)	1.83 (1.08, 2.78)	1.77 (1.06, 2.67)	2.18 (1.26, 3.19)	<0.001
TyG index	8.70 (8.33, 9.11)	8.67 (8.29, 9.06)	8.89 (8.50, 9.37)	<0.001
CTI	8.94 (8.54, 9.38)	8.88 (8.49, 9.31)	9.16 (8.77, 9.71)	<0.001
Left main disease, *n* (%)	140 (5.4)	110 (5.1)	30 (6.5)	0.266
Multivessel disease, *n* (%)	723 (27.7)	573 (26.6)	150 (32.7)	0.010
Chronic total occlusion, *n* (%)	221 (8.5)	169 (7.9)	52 (11.3)	0.020
In-stent restenosis, *n* (%)	105 (4.0)	74 (3.4)	31 (6.8)	0.002
Antiplatelet agent, *n* (%)	2,591 (99.3)	2,137 (99.3)	454 (98.9)	0.359
*β* blocker, *n* (%)	1,354 (51.9)	1,110 (51.6)	244 (53.2)	0.580
ACEI/ARB, *n* (%)	853 (32.7)	696 (32.4)	157 (34.2)	0.477
Statin, *n* (%)	2,533 (97)	2090 (97.2)	443 (96.5)	0.552

BMI, body mass index, CAD, coronary artery disease, PCI, percutaneous coronary intervention, FBG, fasting blood glucose, HbA1c, glycated hemoglobin, TG, triglyceride, TC, total cholesterol, LDL-C, low-density lipoprotein cholesterol, HDL-C, high-density lipoprotein cholesterol, CRP, C-reactive protein, TyG index, triglyceride-glucose index, CTI, C-reactive protein-triglyceride glucose index, ACEI, angiotensin-converting enzyme inhibitors, ARB, angiotensin receptor blocker.

### Kaplan–Meier survival analysis of CTI and outcomes

**Figure 1:**
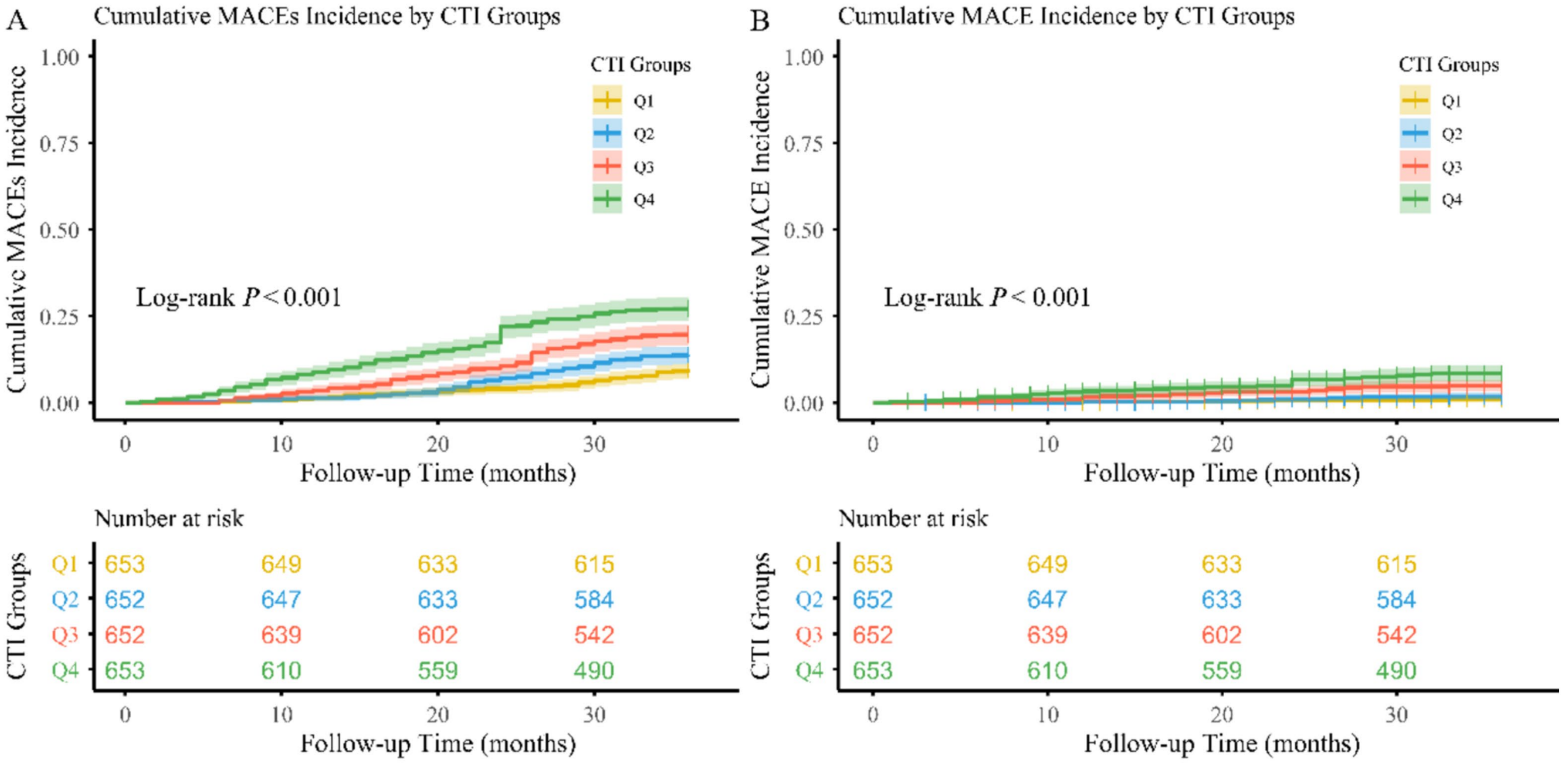
Survival curves of outcomes for different CTI groups. **(A)** The Kaplan–Meier curve shows the association between CTI quartiles and MACEs. **(B)** The Kaplan–Meier curve shows the association between CTI quartiles and MACE. CTI, C-reactive protein-triglyceride glucose index, Q: quartiles, MACEs, major adverse cardiovascular events.

The incidence of MACEs was 17.6% (see Table 2), and it significantly increased with higher CTI quartiles. The Kaplan–Meier curve (see Figure 1) confirmed this association, showing that higher CTI quartiles were significantly associated with an increased incidence of MACEs and MACE (Log-rank *p* < 0.001).

**Table 2:** Outcomes of patients stratified by CTI quartiles.

Variable	Total (*n* = 2,610)	Q1 (*n* = 653) CTI < 8.54	Q2 (*n* = 652) 8.54 ≤ CTI < 8.94	Q3 (*n* = 652) 8.94 ≤ CTI < 9.38	Q4 (*n* = 653) CTI ≥ 9.38	*p*-value
MACEs	459	64 (1.0%)	90 (1.4%)	128 (2.0%)	177 (2.7%)	<0.001
angina pectoris	362	58 (8.9%)	79 (12.1%)	98 (15.0%)	127 (19.5%)	
All-cause mortality	2	0 (0%)	0 (0%)	0 (0%)	2 (0.3%)	
non-fatal acute myocardial infarction	9	0 (0%)	0 (0%)	3 (0.5%)	6 (0.9%)	
ischemia-driven unplanned revascularization	86	6 (0.9%)	11 (1.7%)	27 (4.1%)	42 (6.4%)	

CTI, C-reactive protein-triglyceride glucose index, Q, quartiles, MACEs, Major Adverse Cardiovascular Events.

### Association between CTI and outcomes

We used the Cox proportional hazards regression model to assess the association between CTI and outcomes (see Table 3). In Model 1, without adjusting for covariates, CTI was significantly associated with an increased hazard ratio for MACEs (HR: 2.23, 95% CI: 1.95–2.54, *p* < 0.001) and MACE (HR: 3.83, 95% CI: 2.92–5.03, *p* < 0.001). In Model 2, after adjusting for age, BMI, smoking, and diabetes, CTI remained significantly associated with both MACEs (HR: 2.09, 95% CI: 1.83–2.40, *p* < 0.001) and MACE (HR: 3.30, 95% CI: 2.49–4.37, *p* < 0.001). In Model 3, after further controlling for age, BMI, smoking, previous CAD, previous PCI, hypertension, diabetes, chronic kidney disease, peripheral artery disease, HbA1c, LDL-C, multivessel disease, chronic total occlusion, and in-stent restenosis, CTI remained significantly associated with both MACEs (HR: 2.01, 95% CI: 1.73–2.31, *p* < 0.001) and MACE (HR: 3.01, 95% CI: 2.21–4.09, *p* < 0.001).

**Table 3:** Cox proportional hazards regression analysis of CTI and outcomes.

Variable	Model 1		Model 2			Model 3			
	HR (95% CI)	*p*-value	*P* for trend	HR (95% CI)	*p*-value	*P* for trend	HR (95% CI)	*p*-value	*P* for trend
MACEs			<0.001			<0.001			<0.001
Continuous	2.23 (1.95–2.54)	<0.001		2.09 (1.83–2.40)	<0.001		2.01 (1.73–2.31)	<0.001	
Q1 CTI < 8.54	1.0 (Ref)			1.0 (Ref)			1.0 (Ref)		
Q2 8.54 ≤ CTI < 8.94	1.44 (1.05–1.99),	0.025		1.40 (1.02–1.93)	0.039		1.28 (0.93–1.77)	0.129	
Q3 8.94 ≤ CTI < 9.38	2.14 (1.59–2.89)	<0.001		2.12 (1.57–2.87)	<0.001		1.95 (1.43–2.64)	<0.001	
Q4 CTI ≥ 9.38	3.20 (2.41–4.26)	<0.001		2.92 (2.19–3.91)	<0.001		2.55 (1.89–3.45)	<0.001	
MACE			<0.001			<0.001			<0.001
Continuous	3.83 (2.92–5.03)	<0.001		3.30 (2.49–4.37)	<0.001		3.01 (2.21–4.09)	<0.001	
Q1 CTI < 8.54	1.0 (Ref)			1.0 (Ref)			1.0 (Ref)		
Q2 8.54 ≤ CTI < 8.94	1.87 (0.69–5.05)	0.218		1.76 (0.65–4.77)	0.264		1.46 (0.54–3.95)	0.461	
Q3 8.94 ≤ CTI < 9.38	5.28 (2.20–12.68)	<0.001		4.95 (2.06–11.93)	<0.001		3.79 (1.56–9.19)	0.003	
Q4 CTI ≥ 9.38	9.43 (4.04–21.99)	<0.001		7.54 (3.21–17.76)	<0.001		5.06 (2.11–12.14)	<0.001	

Model 1, Unadjusted. Model 2, Adjusted for age, BMI, smoking, and diabetes. Model 3, Adjusted for age, BMI, smoking, previous CAD, previous PCI, hypertension, diabetes, chronic kidney disease, peripheral artery disease, HbA1c, LDL-C, multivessel disease, chronic total occlusion, and in-stent restenosis. CTI, C-reactive protein-triglyceride glucose index, Q, quartiles, MACEs, Major Adverse Cardiovascular Events, HR, Hazard Ratio, CI, Confidence Interval.

To further investigate the categorical effect of CTI, we divided CTI into quartiles (Q1–4). In Model 1, compared to Q1, the risk of MACEs was significantly higher in Q2, Q3, and Q4, with HRs (95% CI) of 1.44 (1.05–1.99), 2.14 (1.59–2.89), and 3.20 (2.41–4.26), respectively. Regarding MACE risk, the HRs for Q3 and Q4 were 5.28 (2.20–12.68) and 9.43 (4.04–21.99), respectively. In Model 2, after adjusting for age, BMI, smoking, and diabetes, Q2, Q3, and Q4 remained associated with a higher risk of MACEs, with corresponding HRs (95% CI) of 1.40 (1.02–1.93), 2.12 (1.57–2.87), and 2.92 (2.19–3.91), while the HRs (95% CI) for MACE risk were 4.95 (2.06–11.93) and 7.54 (3.21–17.76) for Q3 and Q4, respectively. In Model 3, after further adjusting for various clinical covariates, Q3 and Q4 still showed significantly higher MACEs risk, with HRs (95% CI) of 1.95 (1.43–2.64) and 2.55 (1.89–3.45), respectively, and the HRs (95% CI) for MACE risk were 3.79 (1.56–9.19) and 5.06 (2.11–12.14) for Q3 and Q4, respectively.

**Figure 2:**
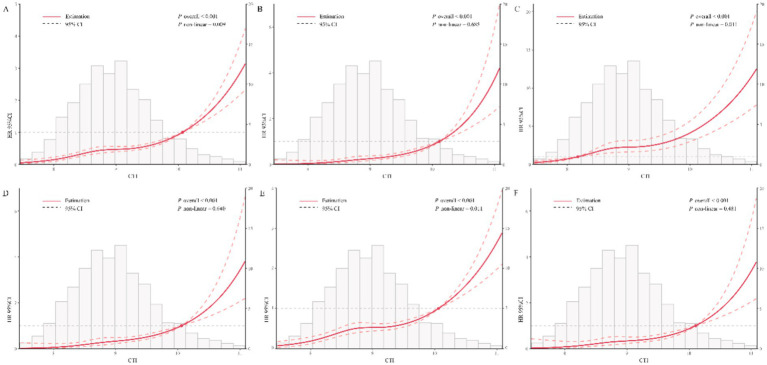
RCS analysis of CTI and outcomes. **(A,B)** Unadjusted. **(C,D)** Adjusted for age, BMI, smoking, and diabetes. **(E,F)** Adjusted for age, BMI, smoking, previous CAD, previous PCI, hypertension, diabetes, chronic kidney disease, peripheral artery disease, HbA1c, LDL-C, multivessel disease, chronic total occlusion, and in-stent restenosis. CTI and MACEs **(A,C,E)**, CTI and MACE **(B,D,F)**. CTI, C-reactive protein-triglyceride glucose index, HR, hazard ratio, CI, confidence interval, RCS, restricted cubic splines.

We further explored the dose–response relationship between CTI and outcomes using restricted cubic splines (see Figure 2). In all three models, we found a significant nonlinear dose–response relationship between CTI and MACEs (*P* nonlinear = 0.009, *P* nonlinear = 0.011, *P* nonlinear = 0.011). However, no significant nonlinear relationship was found between CTI and MACE, with *P* nonlinear values of 0.685, 0.640, and 0.481 in the three models, none of which reached statistical significance.

**Figure 3:**
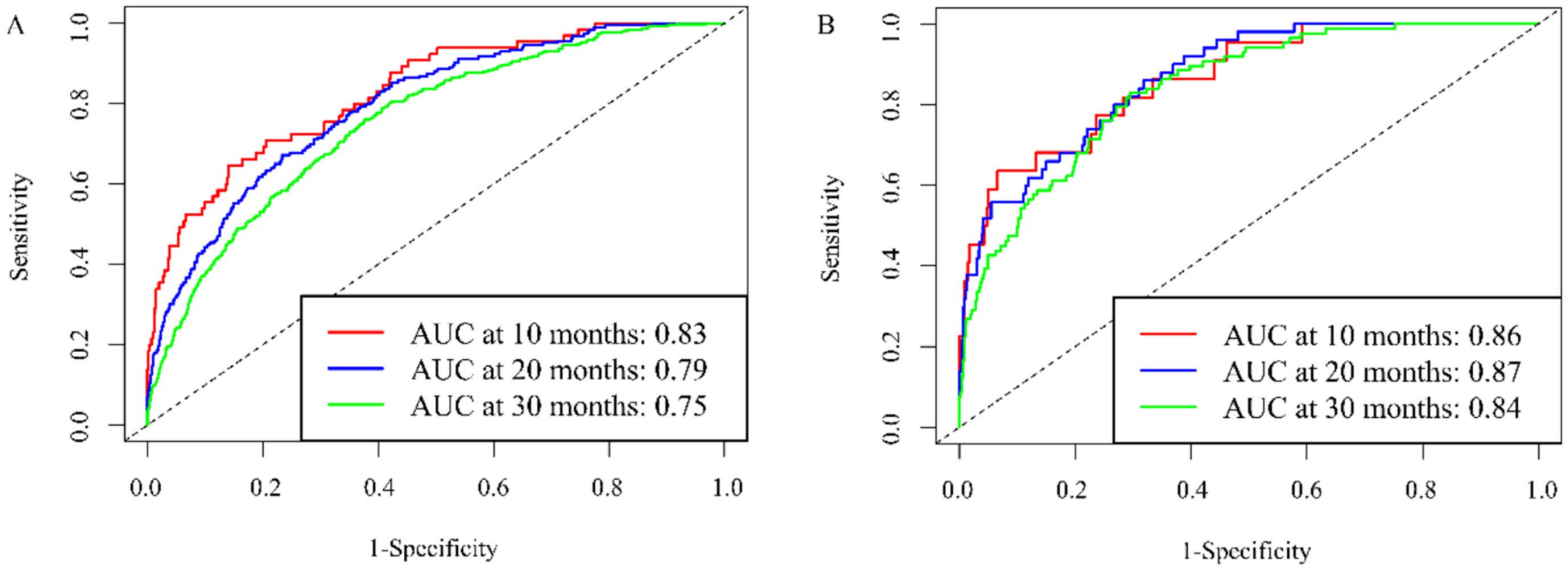
ROC curve between CTI and outcomes. **(A)** ROC curve between CTI and MACEs. **(B)** ROC curve between CTI and MACE. Adjusted for age, BMI, smoking, previous CAD, previous PCI, hypertension, diabetes, chronic kidney disease, peripheral artery disease, HbA1c, LDL-C, multivessel disease, chronic total occlusion, and in-stent restenosis. CTI, C-reactive protein-triglyceride glucose index, ROC, receiver operating characteristic, AUC, area under the curve.

The optimal CTI cut-off values determined by the Youden index were 8.92 for MACEs (sensitivity: 69.1%, specificity: 51.7%) and 8.93 for MACE (sensitivity: 84.5%, specificity: 50.4%; Supplementary Table S1). DeLong tests confirmed that CTI achieved significantly higher areas under the curve (AUCs) than CRP alone and the TyG index alone for both MACEs and MACE (all *p* < 0.05; Supplementary Table S1), indicating that the combined index offers superior risk discrimination compared with either marker in isolation. Given this, we further evaluated the predictive ability of CTI for outcomes using time-dependent ROC curves (see Figure 3). The AUC was positively correlated with CTI’s predictive ability; the larger the AUC, the stronger the predictive ability. After fully adjusting for covariates, ROC curves were plotted at 10, 20, and 30 months as time points. The results showed that the AUC for predicting MACEs with CTI were 0.83, 0.79, and 0.75, while the AUC for predicting MACE were 0.86, 0.87, and 0.84.

### The relationship between the CTI and MACEs using threshold effect analysis

Through threshold effect analysis, we further explored the relationship between CTI and MACEs (see Table 4). In the Cox regression model fully adjusted for covariates, the threshold for CTI was 10.06. When CTI was below this value, each 1 standard deviation increase in CTI was associated with an 87% increased risk of MACEs (HR = 1.87, 95% CI = 1.53–2.28, *p* < 0.001). However, when CTI was at or above 10.06, the HR increased to 2.28 (95% CI: 1.02–5.09, *p* = 0.045). Furthermore, the likelihood ratio for the segmented Cox regression model was <0.001, further supporting the differential impact of CTI on MACEs at different levels.

**Table 4:** The relationship between CTI with MACEs using threshold effect analysis.

Variable	Adjusted HR (95% CI)	*p*-value
Cox regression model	2.01 (1.73–2.31)	<0.001
Segmented Cox regression model		
Inflection point	10.06	
<10.06	1.87 (1.53–2.28)	<0.001
≥10.06	2.28 (1.02–5.09)	0.045
Log-likelihood ratio		<0.001

Adjusted for age, BMI, smoking, previous CAD, previous PCI, hypertension, diabetes, chronic kidney disease, peripheral artery disease, HbA1c, LDL-C, multivessel disease, chronic total occlusion, and in-stent restenosis. CTI, C-reactive protein-triglyceride glucose index, HR, Hazard Ratio, CI, Confidence Interval, MACEs, Major Adverse Cardiovascular Events.

### Subgroup analysis of CTI on outcomes

We performed a subgroup analysis to examine whether the association between increased CTI and outcomes remained consistent across different groups defined by age, gender, smoking, overweight, diabetes, and hypertension (see Table 5). After fully adjusting for covariates, the relationship between CTI and outcomes remained similar in most subgroups. However, in patients aged <65 years, increased CTI was not associated with a higher risk of MACE. Additionally, significant interactions were observed between CTI and MACEs for both age and gender (all *P* for interaction <0.05), and between CTI and MACE for age (*P* for interaction <0.05).

**Table 5:** Association between CTI and outcomes in different subgroups.

Subgroup	*n*	MACEs			MACE		
		HR (95% CI)	*p*-value	*P* for interaction	HR (95% CI)	*p*-value	*P* for interaction
Age				0.016			0.034
<65 years	1,161 (44.5)	1.54 (1.16–2.06)	0.003		1.43 (0.68–3.00)	0.348	
≥ 65 years	1,449 (55.5)	2.23 (1.89–2.62)	<0.001		3.44 (2.44–4.85)	<0.001	
Gender				0.017			0.127
Female	1,011 (38.7)	1.73 (1.38–2.18)	<0.001		2.44 (1.47–4.04)	<0.001	
Male	1,599 (61.3)	2.26 (1.89–2.71)	<0.001		3.61 (2.43–5.38)	<0.001	
Smoking				0.192			0.781
Never	2008 (76.9)	2.14 (1.81–2.53)	<0.001		3.21 (2.22–4.64)	<0.001	
Current/Past	602 (23.1)	1.77 (1.36–2.31)	<0.001		2.87 (1.63–5.04)	<0.001	
Overweight				0.570			0.070
<25 kg/m^2^	1,389 (53.2)	2.03 (1.67–2.46)	<0.001		3.58 (2.29–5.59)	<0.001	
≥ 25 kg/m^2^	1,221 (46.8)	2.06 (1.68–2.53)	<0.001		2.61 (1.71–4.01)	<0.001	
Diabetes				0.133			0.812
No	1778 (68.1)	1.82 (1.48–2.24)	<0.001		3.05 (1.81–5.13)	<0.001	
Yes	832 (31.9)	2.05 (1.68–2.50)	<0.001		2.53 (1.73–3.70)	<0.001	
Hypertension				0.172			0.448
No	963 (36.9)	1.56 (1.18–2.05)	0.002		3.89 (2.10–7.20)	<0.001	
Yes	1,647 (63.1)	2.23 (1.89–2.63)	<0.001		2.96 (2.06–4.26)	<0.001	

Adjusted for age, BMI, smoking, previous CAD, previous PCI, hypertension, diabetes, chronic kidney disease, peripheral artery disease, HbA1c, LDL-C, multivessel disease, chronic total occlusion, and in-stent restenosis. CTI, C-reactive protein-triglyceride glucose index, HR, Hazard Ratio, CI, Confidence Interval, MACEs, Major Adverse Cardiovascular Events.

### Sensitivity analysis

We performed a sensitivity analysis using three different models to assess the stability and consistency of the study results, as shown in Table 6. After excluding participants with Smoking, Previous stroke, chronic kidney disease, and peripheral artery disease, the remaining 1,653 participants were included in the analysis. The results showed that the positive association between CTI and MACEs remained stable across multiple models. After fully adjusting for covariates, the independent positive association between CTI and MACEs persisted (HR = 1.96, 95% CI = 1.61–2.38). When CTI was converted into a categorical variable, with the first quartile (CTI < 8.55) as the reference, the HR (95% CI) for Q3 (8.95 ≤ CTI < 9.37) was 2.43 (1.58–3.73), and the HR (95% CI) for Q4 (CTI ≥ 11.60) was 2.78 (1.81–4.29).

**Table 6:** Sensitivity analysis between CTI and MACEs.

Variable	Model 1			Model 2			Model 3		
	HR (95% CI)	*p*-value	*P* for trend	HR (95% CI)	*p*-value	*P* for trend	HR (95% CI)	*p*-value	*P* for trend
MACEs			<0.001			<0.001			<0.001
Continuous	2.21 (1.84–2.65)	<0.001		1.98 (1.64–2.39)	<0.001		1.96 (1.61–2.38)	<0.001	
Q1 CTI < 8.55	1.0 (Ref)			1.0 (Ref)			1.0 (Ref)		
Q2 8.55 ≤ CTI < 8.95	1.61 (1.02–2.55),	0.041		1.52 (0.96–2.41)	0.072		1.46 (0.92–2.32)	0.106	
Q3 8.95 ≤ CTI < 9.37	2.65 (1.73–4.04)	<0.001		2.48 (1.62–3.79)	<0.001		2.43 (1.58–3.73)	<0.001	
Q4 CTI ≥ 11.60	3.47 (2.30–5.24)	<0.001		2.93 (1.93–4.46)	<0.001		2.78 (1.81–4.29)	<0.001	

Model 1, Unadjusted. Model 2, Adjusted for age, BMI, smoking, and diabetes. Model 3, Adjusted for age, BMI, smoking, previous CAD, previous PCI, hypertension, diabetes, chronic kidney disease, peripheral artery disease, HbA1c, LDL-C, multivessel disease, chronic total occlusion, and in-stent restenosis. CTI, C-reactive protein-triglyceride glucose index, Q, quartiles, MACEs, Major Adverse Cardiovascular Events, HR, Hazard Ratio, CI, Confidence Interval.

## Discussion

This retrospective study examined the relationship between CTI and adverse outcomes following PCI. Our findings show that patients with higher CTI levels had worse outcomes, with the risk of MACEs and MACE increasing progressively with higher CTI quartiles. A non-linear relationship was observed between CTI and the risk of MACEs, while a linear relationship was found between CTI and the risk of MACE. Notably, we identified a threshold effect: when CTI exceeded 10.06, the risk of MACEs rose significantly, indicating a critical level of metabolic and inflammatory stress. Once this threshold is crossed, the body’s homeostatic mechanisms may be overwhelmed, leading to more pronounced pathophysiological disturbances. Subgroup analyses confirmed that the association between CTI and outcomes remained consistent across most subgroups, further supporting its relevance in diverse populations. As such, CTI holds potential as a biomarker that can provide valuable information for postoperative risk stratification.

Inflammation and insulin resistance play pivotal roles in the residual risk of cardiovascular disease. Both are independent cardiovascular risk factors, and their synergistic interaction contributes to the onset and progression of cardiovascular metabolic diseases. CRP is a nonspecific inflammatory marker synthesized in the liver in response to the secretion of various pro-inflammatory cytokines. CRP plays a key role in the pathophysiology of cardiovascular diseases by promoting the development of atherosclerosis and vascular dysfunction, and it is widely recognized as an independent predictor of cardiovascular adverse events (17). Elevated CRP levels are associated with an increased risk of adverse cardiovascular outcomes, including myocardial infarction, stroke, and heart failure (18, 19). Buckley et al. demonstrated that, after comprehensive adjustment for traditional risk factors, elevated CRP levels (>3 mg/L) were independently associated with a 58% increased risk of coronary heart disease (20). Inflammation is a crucial factor in the initiation and progression of atherosclerosis, a process that involves multiple inflammatory cells. An imbalance between pro-inflammatory and anti-inflammatory processes may lead to advanced atherosclerosis (21). Wada and Shitara examined the prognostic value of high-sensitivity C-reactive protein (hs-CRP) in predicting adverse outcomes following PCI (22, 23), with findings consistent with those of Ndrepepa et al. (24). Oemrawsingh et al. studied 486 PCI patients and found that elevated hs-CRP levels at the time of the procedure were strong predictors of 10-year mortality and myocardial infarction risk (25).

In patients with non-ST-segment elevation myocardial infarction, a higher TyG index was independently associated with the severity of CAD and MACEs (HR = 1.88, 95% CI 1.13–3.12) (26). A study involving 986 acute coronary syndrome patients who underwent PCI also demonstrated that elevated TyG index levels were associated with an increased risk of MACEs (27). Additionally, Chen et al. explored the relationship between the TyG index and recurrent revascularization after PCI, finding that a higher TyG index was independently associated with recurrent vascular reconstruction due to in-stent restenosis (28). Another study involving 1,654 patients with in-stent restenosis confirmed that elevated TyG index levels were associated with an increased risk of MACEs (29). The predictive value of the TyG index for adverse cardiovascular events has been attributed to its reflection of insulin resistance. Firstly, insulin resistance-induced dysregulation of glucose and lipid metabolism can lead to inflammation and oxidative stress, thereby contributing to the development of atherosclerosis (30–32). Secondly, insulin resistance is linked to structural and functional damage to the arterial wall, including endothelial dysfunction, impaired vasodilation, increased arterial stiffness, thickening of the intima-media, and coronary artery calcification, all of which are strong predictors of future cardiovascular events (33, 34). Furthermore, insulin resistance may enhance platelet reactivity, increasing platelet aggregation and the expression of platelet A2-dependent tissue factor, ultimately promoting thrombosis and inflammation (35). A study by Ma et al. in 776 patients with type 2 diabetes and acute coronary syndrome undergoing PCI found that the TyG index was associated with all-cause mortality, non-fatal stroke, non-fatal myocardial infarction, and unplanned repeat vascular revascularization (36). Similarly, Zhu et al. found that in acute coronary syndrome patients undergoing PCI, an elevated TyG index was independently and positively correlated with in-stent restenosis (37).

The interaction between inflammation and insulin resistance is a key driving force in atherosclerosis. Chronic inflammation can impair insulin sensitivity, while insulin resistance, in turn, exacerbates the inflammatory response. This vicious cycle further accelerates the progression of atherosclerosis and its complications. Based on these findings, our study identified CTI as an independent predictor of PCI prognosis. Subgroup analyses further confirmed the stability of the association between CTI and prognostic risk, with this association persisting across most subgroups. CTI integrates two dimensions—insulin resistance and systemic inflammation—into a single marker, reflecting the synergistic damage caused by metabolic and inflammatory pathways. The comprehensive assessment system based on insulin resistance and inflammation markers offers a more holistic view of the dynamic interplay between metabolism and inflammation, providing more accurate insights for disease diagnosis and management. In a study of 8,679 participants from NHANES, higher CTI values were significantly associated with increased cardiovascular disease mortality and incidence (38). In the China Health and Retirement Longitudinal Study, a study by Ou et al. (39) involving 17,705 middle-aged and older adults found that higher CTI levels were significantly associated with cardiovascular disease and mortality risk, and a linear relationship was observed between CTI and all-cause mortality.

### Limitations

This study has several limitations that should be considered. First, as a single-center retrospective study, causal inference between CTI levels and prognosis cannot be established, and the findings may be subject to selection bias, limiting their geographical and demographic representativeness. External validation in independent cohorts from different clinical centers or diverse geographical regions is warranted to confirm the generalizability of the CTI index across broader populations. Second, although potential confounders were thoroughly adjusted for in the analysis, the retrospective design may still introduce residual or unmeasured confounders. Third, this study only included patients without acute myocardial infarction, which may have led to an underestimation of the incidence of MACEs in the current analysis, thus introducing selection bias and limiting the applicability of the findings to a broader CAD population. Additionally, the relatively short follow-up period may have failed to capture long-term risk variations. Fourth, although discharge medication prescriptions were collected and showed comparable rates between groups (Table 1), long-term medication adherence during follow-up was not systematically monitored, which may introduce residual confounding unrelated to the CTI’s predictive value. Future prospective studies should incorporate serial adherence assessments to verify that CTI remains an independent predictor regardless of treatment intensity. Fifth, detailed procedural characteristics, including the specific generation of drug-eluting stents and anatomical complexity scores such as the SYNTAX score, were not available for analysis. Subgroup analyses stratified by stent type and lesion complexity would help clarify whether the prognostic value of CTI remains consistent across different interventional strategies and anatomical scenarios. Finally, CTI was assessed only at baseline, and serial measurements of CRP and the TyG index during follow-up were not collected, precluding the evaluation of intra-individual changes in CTI over time and their prognostic implications. Future studies with repeated biomarker assessments could employ time-dependent Cox regression or landmark analysis to determine whether a reduction in CTI over time is associated with improved survival, thereby capturing the dynamic relationship between metabolic-inflammatory status and long-term prognosis.

## Conclusion

In conclusion, pre-PCI CTI levels are significantly associated with clinical outcomes and may serve as a valuable marker for secondary prevention in CAD patients following PCI.

## Data Availability

The original contributions presented in the study are included in the article/Supplementary material, further inquiries can be directed to the corresponding authors.
