# Supplementary material for: Association between the C-reactive protein-triglyceride-glucose index and major adverse cardiovascular events in patients undergoing percutaneous coronary intervention
Source: Front Med (Lausanne). 2026 May 29;13:1858059. doi: 10.3389/fmed.2026.1858059 (PMC13259643; doi:10.3389/fmed.2026.1858059)
Supplement: Supplementary file 1 [file Table_1.docx]

**Supplementary Table S1. DeLong test comparing the predictive performance of CTI with CRP and the TyG index for MACEs and MACE.**

| Endpoint | Variables | AUC | ΔAUC  (95% CI) | Z | *P* value | Optimal cut-off | Sensitivity | Specificity |
| --- | --- | --- | --- | --- | --- | --- | --- | --- |
| MACEs | CTI | 0.643 | — | — | — | 8.92 | 69.1% | 51.7% |
|  | CRP | 0.590 | 0.053 (0.019–0.087) | 3.034 | 0.002 | — | — | — |
|  | TyG | 0.614 | 0.029 (0.017–0.041) | 4.757 | <0.001 | — | — | — |
| MACE | CTI | 0.729 | — | — | — | 8.93 | 84.5% | 50.4% |
|  | CRP | 0.566 | 0.163 (0.093–0.232) | 4.583 | <0.001 | — | — | — |
|  | TyG | 0.705 | 0.024 (0.002–0.046) | 2.097 | 0.036 | — | — | — |

Abbreviations: CTI: C-reactive protein-triglyceride glucose index, AUC: Area Under the Curve, CRP: C-reactive protein, TyG index: triglyceride-glucose index, MACEs: Major Adverse Cardiovascular Events, CI: Confidence Interval, ΔAUC, difference in AUC between CTI and the comparator.
